# The Job Leeway Scale: Initial Evaluation of a Self-report Measure of Health-Related Flexibility and Latitude at Work

**DOI:** 10.1007/s10926-023-10095-6

**Published:** 2023-03-27

**Authors:** William S. Shaw, Alicia G. Dugan, Alyssa K. McGonagle, Michael K. Nicholas, Torill H. Tveito

**Affiliations:** 1 University of Connecticut School of Medicine, Farmington, CT USA; 2 University of North Carolina, Charlotte, NC USA; 3 University of Sydney, Sydney, NSW Australia; 4 University of South-Eastern Norway, Horten, Norway

**Keywords:** Work disability, Accommodation, Chronic symptoms, Flexibility, Leeway, Self-management, Organizational support

## Abstract

*Purpose* Evidence suggests that workers manage health-related challenges at work, in part, by using available leeway to perform work differently. The purpose of this study was to evaluate the reliability and validity of the Job Leeway Scale (JLS), a new 18-item self-report questionnaire designed to assess worker perceptions of available flexibility and latitude to manage health-related challenges at work. *Methods* Workers seeking assistance for workplace difficulties due to chronic medical conditions (*n* = 119, 83% female, median age = 49) completed the JLS along with other workplace and health measures. Construct validity was assessed using exploratory factor analysis (EFA), and concurrent validity was assessed by associations with related measures. *Results* Mean item scores ranged from 2.13 to 4.16 within a possible range of 0–6. The EFA supported three underlying factors: organizational leeway (9 items), task leeway (6 items), and staffing leeway (3 items). Internal consistency (alpha) ranged from 0.78 to 0.91 for subscale scores and 0.94 for the total score. The JLS showed moderate correlations with other work outcome measures including work fatigue, self-efficacy, engagement, and productivity. *Conclusion* The JLS is a promising new measure with initial support for its reliability and validity to assess worker beliefs of available flexibility to manage health symptoms at work, and this construct may have organizational implications for worker support and accommodation.

Episodic, intermittent, and chronic health problems are a significant workplace challenge in an aging population [1–3], and these fluctuating symptoms put workers at risk for sickness absence, job loss, early retirement, and permanent work disability [1–5]. Employers are required to provide reasonable job accommodations when requested, but there is evidence that workers also find ways to leverage existing flexibility during symptom flare-ups to get their work done without having to take time off work [6–8]. Workers can use self-regulatory processes to adjust their day-to-day work and schedules to overcome intermittent symptoms only if adequate leeway is available based on job demands and employer policies [6]. Related and overlapping concepts in the literature include informal accommodation strategies [9], margin of maneuver [10], job flexibility [8, 11, 12], adjustability [10], natural organizational support [13], and iterative communication-support processes between workers and their supervisors [14, 15]. Job flexibility has, in general, been associated with improved quality of working life for employees with chronic disease [4, 11], but the concept of job leeway may be especially salient for workers with episodic health problems. In this context, we define *job leeway* as the margins of freedom, variation, and tolerance available to workers to self-regulate work activities while self-managing day-to-day symptom fluctuations. Currently, there are no self-report measures of job leeway that might identify its impact on disability outcomes.

One aspect of job leeway is the adjustability afforded by the work itself. School bus drivers have little leeway to alter their work environment, schedule, or tasks to reduce the impact of pain or other functional limitations while working. In contrast, an office worker doing computer work from home has considerably more leeway to modify the choice, ordering, and scheduling of job tasks and to alter the work environment and daily work habits to accommodate intermittent health challenges. Such sources of leeway might be critical for workers to manage recurring symptoms that are unpredictable and pose intermittent functional limitations. Other aspects of job leeway may be afforded by organizational policies and standards of individual employers or worksites. Some employers may choose to allow flexibility and variability, and this has benefits for workers with recurring health problems to fulfill their assigned roles. For example, permitting temporary leeway to production workers can lessen biomechanical load and preserve health for all workers, especially older workers [16]. Other employers may opt for less flexibility to support operational efficiency, maintain product quality, or minimize perceived safety hazards. Workers with intermittent or episodic health symptoms may be less suited to these employment settings because they lack the necessary leeway to alter work schedules and habits on a day-to-day basis in response to fluctuating symptoms.

While formal requests for job accommodation are the standard procedure for employers to adjudicate necessary adjustments for workers with disabilities, this formal administrative process can be cumbersome when needs are intermittent or episodic [15], when accommodations involve changes to organizational processes or occasional co-worker assistance [9], or when symptoms do not translate easily into discrete physical workload alterations [17]. There is also evidence that some workers fail to request accommodations because of negative experiences or concerns related to privacy, stigma, or disclosure [18, 19]. In one study of 408 US workers with disabilities, informal accommodation strategies were more frequently reported (52.7%) than were formal requests for accommodation under the regulatory provisions of the Americans with Disabilities Act (ADA) [9]. Some transient problems with workplace function may be overcome if workers can take advantage of existing job leeway afforded by their work tasks, organizational policies, and supervisory communication.

Given the large proportion of working-age adults with at least one chronic medical condition [1–3], there is a need to better understand the range of organizational factors and job characteristics that support the health coping and self-management strategies of these employees during working hours. To improve the conceptualization of job leeway and to provide a new self-report measure of this construct for research applications, the authors developed the Job Leeway Scale (JLS), an 18-item questionnaire. The initial item pool was generated from focus groups of workers with chronic musculoskeletal pain [6]. In this report, we present a pilot administration of the quantitative measure in a sample of workers with chronic illness who were seeking assistance to manage symptoms on the job. This provided an opportunity for an initial psychometric evaluation of the JLS with respect to its reliability (internal consistency), construct validity (factor structure), and concurrent validity (association with related validated measures).

## Method

### Participants

Participants were employees with chronic health conditions who expressed interest to participate in a group education program designed to improve coping and function at work [20]. Most were recruited from four worksites in the northeastern USA, including two large hospitals, a regional outpatient health care system, and a high-technology manufacturing firm. Eligible participants were full-time workers (> 20 h per week) of at least 18 years of age. All participants reported at least one chronic health condition lasting more than 6 months that was beginning to present workplace challenges (see list of conditions in Results, below). To avoid unnecessary health disclosures in the workplace, participants were not required to indicate specific medical diagnoses to qualify for the study, but this information was shared by consenting participants later in a confidential research survey. Reading and speaking in English language were inclusionary criteria because of the nature of the interactive group intervention program. We also excluded workers who were already planning to retire or change jobs in the next 12 months and those who were unable to participate in group workshops before work, after work, or during lunch hours. The group intervention program and surveys were conducted outside regular working hours, but employers supported the program by advertising it through newsletters, posters, and flyers, and by providing a private, on-site location for group meetings.

### Procedures

Detailed study procedures, including steps of the intervention design process and a detailed list of survey measures, are described in the published study protocol [20] and clinical register (clinicaltrials.gov, #NCT01978392). The study was publicized through posted flyers, newsletter entries, and email announcements sent to the entire workforce. On-site occupational health and safety staff also referred to the study workers who had experienced multiple disability absences or expressed concerns about on-going health challenges. The nature of the intervention was a 10-session group educational workshop aimed at improving the pain and illness self-management strategies of workers, with a special focus on strategies that could be implemented during working hours. Group comparisons of intervention outcomes for the randomized trial have been published previously [21].

Interested workers contacted a project coordinator who provided information about the study, answered questions, screened participants, obtained informed consent, and administered the baseline survey. Participants randomized to the treatment group participated in a 10-session group intervention program designed to improve workplace support and self-management, but the current study pertains only to data collected as baseline measures. All procedures were reviewed and approved by the institutional review board of the Liberty Mutual Research Institute for Safety, where the lead author was affiliated at the time of data collection, and all study participants provided written informed consent.

### Measures

#### The Job Leeway Scale

The original version of the Job Leeway Scale (JLS) was comprised of 18 items that were generated by the authors based on the results of a qualitative focus group study among workers with chronic musculoskeletal pain [6]. We strove to include the full range of leeway elements expressed in focus groups and represented among primary themes generated by qualitative analysis of transcripts [6]. Though the original study focused on pain, it was the authors’ intent to make the JLS relevant across other diagnostic conditions that commonly pose challenges in the workplace. The rationale for the JLS scale was to provide a reliable and valid self-report instrument that assessed perceptions of available job leeway during times of symptom flare-ups or on days of increased functional difficulties. The format of the scale asked respondents to consider the flexibility offered by their jobs when not feeling well and to report these elements of job leeway on a 7-point Likert scale from 0 (completely disagree) to 6 (completely agree). Items were designed to reflect multiple sources of leeway (supervisor, working group, human resources, nature of job tasks, personal self-management strategies) and to be appropriate for most job types and employment settings. The lead-in wording for each item (“When I’m not feeling well, I can….”) was chosen to apply to a wide range of possible health circumstances. A total score was computed as an average of all 18 items (possible range from 0 to 6). One item (“having to keep working like everyone else”) was phrased in the negative direction. A copy of the JLS is included as Appendix A.

### Concurrent Measures

Related workplace constructs were administered simultaneously with the JLS to assess evidence for concurrent validity of the JLS. In this secondary analysis, the choice of concurrent measures was dictated by the content and goals of the intervention program that was the focus of the study, including primary and secondary outcome measures and covariates. The two primary outcome measures were work engagement and work limitations, and secondary outcomes included self-efficacy and fatigue [20]. A measure of working conditions served as a potential covariate when evaluating the effects of the intervention program [20]. Each of these measures and their psychometric properties are described below.

#### Working Conditions

The Areas of Worklife Survey (AWS) [22] provided a brief measure of general working conditions including workload, organizational support, and psychosocial working environment [23, 24]. A Principal Components Analysis of the 28-item AWS provided evidence for six workplace dimensions: (1) workload; (2) control; (3) reward; (4) community; (5) fairness; and (6) values [25]. Internal consistency (alpha) of the six subscales ranges from 0.67 (workload) to 0.83 (control) [25]. Test-retest correlations vary from 0.51 (reward) to 0.62 (workload). External validity of the AWS is supported by the correlation of subscales with workers’ voluntary complaints [25]. Respondents rated their level of agreement on a 5-point Likert scale from 1 (strongly disagree) to 5 (strongly agree). We chose this scale to test correlations of the JLS with other related workplace constructs, especially that of perceived control, fairness, and values.

#### Worker Fatigue

The 20-item Occupational Fatigue, Exhaustion, Recovery (OFER) scale [26] assesses the degree to which job activities produce acute fatigue, deplete available energy for after-work activities, and reduce the ability to engage in pleasurable activities after work. Respondents rated their level of agreement on a 7-point Likert scale from 1 (completely disagree) to 7 (completely agree). The measure has good test-retest reliability, and confirmatory factor analyses have shown strong support for its construct validity [27]. We chose this measure based on our prior qualitative work, that showed a high level of exhaustion and inactivity at the end of the workday among workers with chronic health conditions [6].

#### Work Self-efficacy

The 19-item Return-to-work Self-Efficacy (RTWSE-19) scale was used to assess the certainty with which individuals felt able to overcome health-related workplace barriers for managing symptoms at work, obtaining help from others, and keeping up with job demands [28]. Respondents report on a scale from 0 to 10 their level of confidence to manage or overcome potential workplace challenges. This scale was designed to provide a measure of the interaction between workplace barriers and personal self-management and problem-solving efforts. The internal consistency (alpha) for three factor analyzed subscales (meeting job demands, modifying job tasks, and communicating needs to others) vary from 0.81 to 0.98 [28]. Evidence for validity of the RTWSE-19 is its prediction of return-to-work and other important work disability outcomes [29, 30].

#### Productivity Loss

The Work Limitations Questionnaire (WLQ) is a 25-item self-report questionnaire that was used to assess the degree to which workers experienced limitations at work due to their health [31, 32]. Respondents rated their frequency of difficulty or ability to perform specific job demands including time management, physical demands, mental-interpersonal demands, and output demands. The WLQ responses are on a 5-point scale from 1 (all of the time) to 5 (none of the time). The scale has good internal consistency and has been validated against other health and disability constructs [32]. A Productivity Index score estimates the total percentage loss in work output due to health [33].

#### Work Engagement

The short-form (9-item) version of the Utrecht Work Engagement Scale (UWES) provided a measure of workplace function by assessing the degree to which employees had an energetic sense of connection with their work activities and viewed themselves as able to deal effectively with job demands [34]. The UWES asks respondents to report the frequency of work engagement items on a 7-point scale from 0 (never) to 6 (always or every day). The UWES has good psychometric properties [35] and captures a holistic view of work performance that encompasses elements of vigor, dedication, and absorption.

### Data Analysis

Reliability of the JLS was evaluated by its internal consistency (Cronbach’s alpha). Construct validity was assessed using exploratory factor analysis (EFA, principal components analysis with varimax rotation). Concurrent validity was assessed by examining expected correlations of the JLS with concurrently-administered measures of other workplace constructs without showing complete redundancy. We also tested whether associations with the JLS remained statistically significant after controlling for the known effects of workload and job control. These two covariates were chosen because of their existing well-established role in predicting many workplace outcomes consistent with the Job Demands-Resources model [36]. All analyses were conducted with the SPSS statistical software package for the Social Sciences [37].

## Results

A total of 119 participants (98 female, 20 male, 1 other) provided informed consent and completed the baseline survey before being randomized to intervention arms. The most frequent occupations were administrative assistant (19%), manager/supervisor (17%), data analyst or research assistant (13%), medical assistant (12%), medical technologist (9%), nurse or nursing assistant (7%), and coding or billing specialist (7%). Other less frequent occupations included lab scientists, engineers, teachers, cashiers, assemblers, and counselors.

The most common chronic health conditions were back or neck pain (85%), hand/arm pain (61%), leg or foot pain (55%), migraine or severe headaches (43%), visual problems (31%), gastrointestinal disorders (24%), respiratory disorders (22%), mental health disorders (17%), cardiovascular disease (5%), skin disorders (4%), and diabetes (4%). Participants reported a median of 3 chronic health categories and a mean of 5.7 lost workdays due to health (range 0–70 days) over the prior six months. Demographic characteristics (Table 1) reflect a sample of mostly White, non-Hispanic, middle-aged workers with college degrees and moderate annual income (US$30,000 to $80,000). Female workers were older (mean age of 47.0 versus 41.0 for males) and had been with their current employer longer (*p* < 0.05).

**Table 1:** Participant demographic characteristics

	All participants (*n* = 119)	Males (*n* = 20)	Females (*n* = 99)	*t* or *χ* ^2^	*p*
	M (SD)/*n* (%)	M (SD)/*n* (%)	M (SD)/*n* (%)		
Age	46.0 (12.7)	41.0 (12.9)	47.0 (12.4)	*t* = 1.96	0.05
Chronic health conditions	3.7 (1.9)	3.8 (1.9)	3.7 (1.9)	*t* = 0.31	0.76
Children/dependents at home				*χ* ^2^ = 1.77	0.78
Yes	59 (49.6)	11 (55.0)	48 (48.5)		
No	60 (50.4)	9 (45.0)	51 (51.5)		
Race				*χ* ^2^ = 2.99	0.22
Asian	2 (1.7)	1 (5.0)	1 (1.0)		
Black	7 (5.9)	0 (0.0)	7 (7.1)		
White	108 (90.8)	19 (95.0)	89 (89.9)		
Not reported	2 (1.7)	0 (0.0)	2 (2.0)		
Ethnicity				*χ* ^2^ = 3.12	0.08
Hispanic	4 (3.4)	2 (1.0)	2 (2.0)		
Non-Hispanic	112 (94.1)	18 (90.0)	94 (95.0)		
Not reported	3 (2.5)	0 (0.0)	3 (3.0)		
Marital status				*χ* ^2^ = 1.15	0.77
Never married	25 (21.0)	4 (20.0)	21 (21.2)		
Married/partnered	67 (56.3)	13 (65.0)	54 (54.5)		
Divorced/separated	25 (21.0)	3 (15.0)	22 (22.2)		
Widowed	2 (1.7)	0 (0.0)	2 (2.0)		
Annual income				*χ* ^2^ = 11.47	0.25
$10,000–$29,999	17 (14.3)	3 (15.0)	14 (14.1)		
$30,000–$39,999	27 (22.7)	3 (15.0)	24 (24.2)		
$40,000–$49,999	27 (22.7)	3 (15.0)	24 (24.2)		
$50,000–$59,999	11 (9.2)	3 (15.0)	8 (8.1)		
$60,000–$69,999	10 (8.4)	4 (20.0)	6 (6.1)		
$70,000 or over	26 (21.8)	4 (20.0)	22 (22.2)		
(missing)	1 (0.8)	0 (0.0)	1 (1.0)		
Highest education				*χ* ^2^ = 7.81	0.10
< 12 years	1 (0.8)	1 (5.0)	0 (0.0)		
High school	9 (7.6)	1 (5.0)	8 (8.1)		
Some college	54 (45.4)	6 (30.0)	48 (48.5)		
Bachelor’s degree	27 (22.7)	5 (25.0)	22 (22.2)		
Post-bachelor’s	28 (23.5)	7 (35.0)	21 (21.2)		
With current employer				*χ* ^2^ = 15.29	0.01
0–6 months	8 (6.7)	2 (10.0)	6 (6.1)		
6–12 months	8 (6.7)	3 (15.0)	5 (5.0)		
1–2 years	8 (6.7)	0 (0.0)	8 (8.1)		
2–5 years	34 (28.6)	11 (55.0)	23 (23.2)		
5–10 years	29 (24.4)	1 (5.0)	28 (28.3)		
> 10 years	32 (26.9)	3 (15.0)	29 (29.3)		

Means and standard deviations for each of the 18 initial JLS items are shown in Table 2. The three items with the highest mean scores were: controlling the pacing of work, rotating between job tasks, and taking micro-breaks. The lowest rated JLS items were working from home, finding a more comfortable place to work, and rescheduling some activities for later. The full range of possible responses (from 0 to 6) were utilized on all 18 items, and frequency histograms showed no obvious floor or ceiling effects for any of the individual items. A visual inspection of item-characteristic curves (plots of individual items versus total JLS scores) showed no anomalies that might suggest some items should be dropped due to poor wording or comprehension. Only 3 items on the scale had missing values: “rotate between job tasks” (*n* = 1), “choose easier job tasks” (*n* = 3), and “work from home” (*n* = 2). These items may be less applicable for some occupations.

**Table 2:** Exploratory factor analysis of the Job Leeway Scale (*n* = 114)

Item	Description	M	SD	EFA factor loadings^a^		
				Factor 1	Factor 2	Factor 3
Subscale 1: Organizational leeway						
14	Find a more comfortable place	2.70	1.80	0.79		
10	Can work from home	2.13	2.01	0.76		
18	Leeway to get through the day	3.67	1.98	0.73		
17	Job can be flexible	3.49	2.03	0.71		
11	Reschedule some activities	3.04	2.08	0.67		
16	Take micro-breaks	3.92	2.04	0.62		
15	Dress more comfortably	3.78	2.34	0.61		
7	Choose physical or seated tasks	3.65	2.24	0.61		
5	Physical aspects can be altered	3.30	1.93	0.47		
Subscale 2: Task leeway						
3	Choose easier job tasks	3.62	1.92		0.70	
9	Able to vary my work	3.91	1.96		0.70	
2	Control pacing of my work	4.16	2.00		0.64	
1	Rotate job tasks	4.02	2.12		0.63	
4	Special tools or equipment	3.15	1.82		0.49	
8	Perform like everyone else	3.80	2.44		− 0.48	
Subscale 3: Staffing leeway						
13	Depend on others to help	3.59	1.95			0.88
12	Others can shift to help me	3.08	1.91			0.85
6	Reduce discomfort and stress	3.08	1.76			0.62

^a^Principal components analysis with varimax rotation, loadings > 0.40 shown

**Fig. 1:**
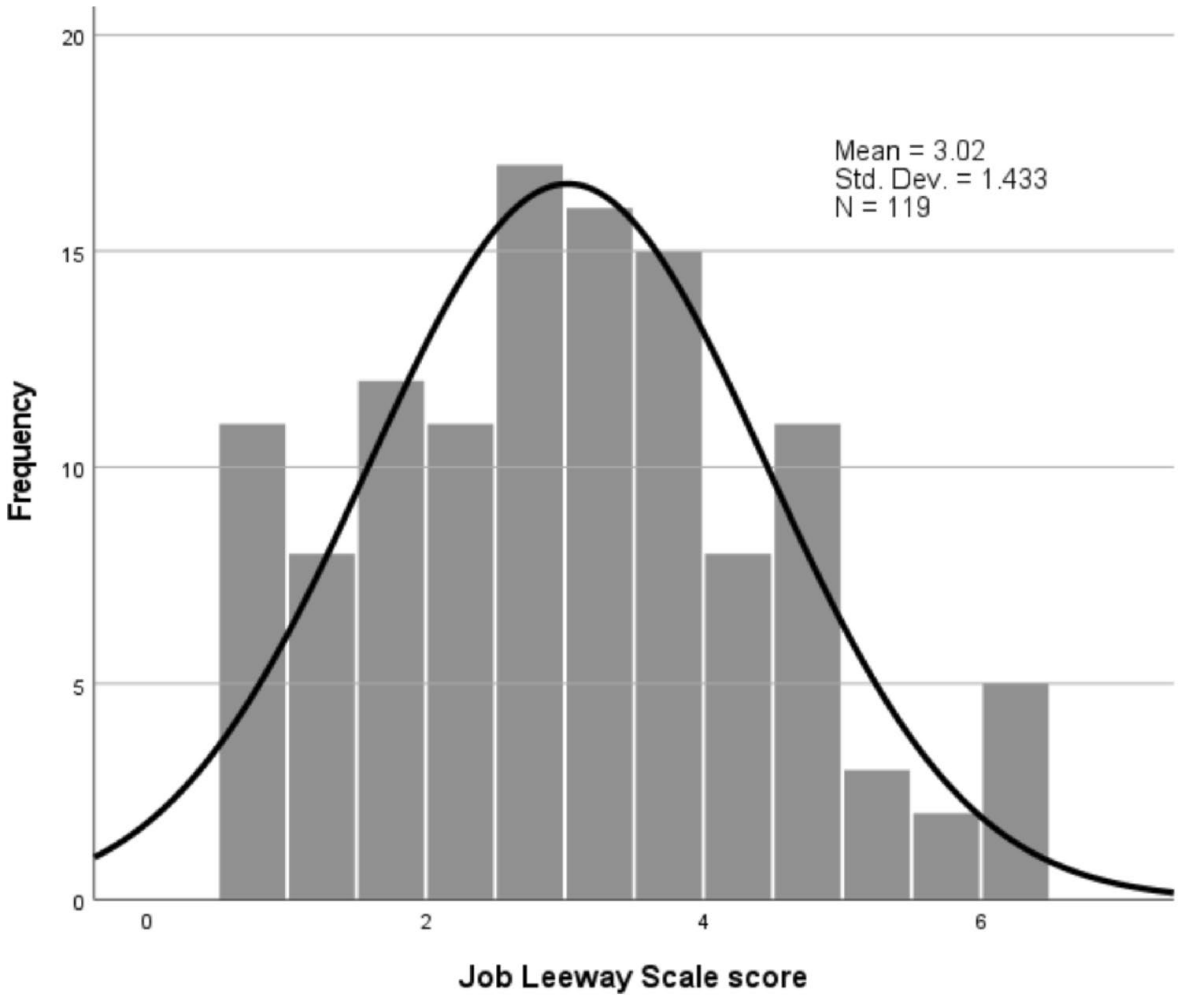
Distribution of total JLS scores with normal distribution (*n* = 119)

After reverse coding the one negatively phrased item (“having to keep working like everyone else”), total JLS scores (an average of all items not missing) ranged from 1.00 to 6.00 (*M* = 3.02, *SD* = 1.43), and tertiles (3 equally sized groups) were defined as low (< 2.4), medium (2.4–3.6), and high (> 3.6). The total score showed no statistically significant associations by age, gender, race, ethnicity, marital status, dependents, or job tenure (t-tests, one-way analyses of variance, or correlations, *p* > .05), but there were significant positive associations of leeway with education (one-way analysis of variance, linear contrast, *F*[1,114] = 6.35, *p* < 0.05), and income (one-way analysis of variance, linear contrast, *F*[1,108] = 7.24, *p* < 0.05). Mean values of leeway by education showed increases from 2.83 for high school, 3.33 for bachelor’s degree, and 3.46 for post-graduate degree. Mean values by income were, for example, 2.60 ($10–20k), 2.82 ($40–50k), 3.09 ($50–60k), and 3.73 ($80–90k). A histogram showing the distribution of total scores is shown in Fig. 1.

Factor analysis (principal components) of the 18 items showed 3 factors with eigenvalues > 1.0, and this factor solution explained 67.7% of the total variance among JLS items. The rotated factor loadings applying a varimax rotation are shown in Table 2. Labels were assigned to factors based on the items loading the highest on each factor. Factor 1 was labeled “organizational leeway” (9 items) because it described elements of leeway related to scheduling, physical environment, breaks, and personal comfort. Factor 2 was labeled “task leeway” (6 items) because it described the variability and modifiability of job tasks. Factor 3 was labeled “staffing leeway” (3 items) because it described the ease of obtaining assistance from others to reduce loads or cope with added stressors. Internal consistency (alpha) for the three scales were 0.91, 0.78, and 0.86, respectively, and alpha was 0.94 for the full-scale total.

Correlations of the JLS total score with concurrent measures are shown in Table 3. For the six subscales of the Areas of Working Life (AWL) survey, correlations with the JLS ranged from 0.15 to 0.61. Only the correlation of leeway with workload did not reach statistical significance (*p* > 0.05). Positive correlations were strongest for the AWL control and reward subscales. The JLS showed significant correlations (*p* < 0.05) with all four workplace outcomes and measures (fatigue, self-efficacy, engagement, and productivity loss), with correlations ranging from − 0.24 (with productivity loss) to 0.59 (with work self-efficacy). To determine if the JLS explained additional variance in work outcomes after controlling for workload and control, we conducted additional multiple regression analyses for each of the four outcome measures (Table 4). These results showed that the JLS explained additional unique variability in fatigue and self-efficacy, but not for engagement or productivity loss. In these analyses, a check for multiple collinearity problems among regressors showed no variance inflation factors exceeding 5.0 (the greatest value was 1.6).

**Table 3:** Correlations of leeway with other workplace variables (*n* = 119)

Scale	(2)	(3)	(4)	(5)	(6)	(7)	(8)	(9)	(10)	(11)
(1) Leeway	0.15	0.61**	0.51**	0.39**	0.29**	0.33**	− 0.34**	0.59**	0.24*	− 0.24*
(2) Workload		0.33**	0.36**	0.36**	0.43**	0.20*	− 0.48**	0.42**	0.24**	− 0.38**
(3) Control			0.53**	0.49**	0.44**	0.39**	− 0.43**	0.60**	0.38**	− 0.44**
(4) Reward				0.65**	0.61**	0.39**	− 0.42**	0.65**	0.49**	− 0.37**
(5) Community					0.50**	0.35**	− 0.39**	0.58**	0.40**	− 0.38**
(6) Fairness						0.40**	− 0.38**	0.41**	0.38**	− 0.23*
(7) Values							− 0.27**	0.39**	0.56**	− 0.21*
(8) Workplace fatigue								− 0.64**	− 0.48**	0.51**
(9) Work self-efficacy									0.45**	− 0.45**
(10) Work engagement										− 0.31**
(11) Productivity loss										

*p < 0.05, **p < 0.01

**Table 4:** Summary of multiple regression analyses explaining job leeway associations with workplace outcomes while controlling for workload and job control (*n* = 119)

Variable	B	SE B	β	*p*
Outcome: Workplace fatigue (OFER)				
Workload (AWS)	− 0.513	0.122	− 0.343	< 0.001
Control (AWS)	− 0.267	0.119	− 0.206	0.027
Job Leeway (JLS)	− 0.174	0.080	− 0.202	0.031
Outcome: Work self-efficacy (RTWSE-19)				
Workload (AWS)	0.252	0.096	0.192	0.010
Control (AWS)	0.429	0.094	0.376	< 0.001
Job Leeway (JLS)	0.220	0.063	0.291	< 0.001
Outcome: Work engagement (UWES)				
Workload (AWS)	0.199	0.124	0.148	0.111
Control (AWS)	0.443	0.121	0.381	< 0.001
Job Leeway (JLS)	− 0.071	0.081	− 0.092	0.382
Outcome: Productivity Loss (WLQ)				
Workload (AWS)	− 1.514	0.515	− 0.264	0.004
Control (AWS)	− 1.750	0.503	− 0.340	< 0.001
Job Leeway (JLS)	− 0.029	0.333	− 0.009	0.931

*OFER* Occupational fatigue, exhaustion, recovery scale, *AWS* Areas of worklife survey, *JLS* Job Leeway Scale, *RTWSE-19* Return-to-work self-efficacy Scale, *UWES* Utrecht Work Engagement Scale, *WLQ* Work limitations questionnaire

## Discussion

This study describes an initial psychometric evaluation of a new self-report measure, the Job Leeway Scale (JLS), created by the authors to assess perceived levels of temporary workplace leeway available to workers with episodic, intermittent, or chronic health problems. The goal or our analyses was to assess reliability (internal consistency), construct validity (factor structure), and concurrent validity (association with related constructs). Job leeway was conceptualized as a potentially important characteristic of jobs and organizations that might improve the ability of workers to stay at work while managing transient symptom fluctuations and intermittent functional challenges.

JLS items were generated by the authors from qualitative findings among workers with chronic health conditions [6]. The JLS asks respondents to indicate the extent to which specific work characteristics can be modified when not feeling well. In this initial evaluation of the JLS, survey respondents used the full range of response options, there were few missing values, and we observed no evidence of poor comprehension or problematic floor or ceiling effects. A plot of the JLS total score showed evidence of a normal distribution, and there were no significant differences by age, gender, or job tenure. Workers with higher education and income reported higher levels of job leeway.

EFA results showed preliminary evidence of three underlying subdomains of the JLS: leeway with respect to organizational policies, leeway inherent in job tasks, and leeway related to available staffing support and assistance. Future studies might repeat EFA results of conduct a confirmatory factor analysis in a larger and more diverse sample of workers. Internal consistency of the three subscales was moderate to high, and there was high internal consistency for the JLS overall score.

In support of its concurrent validity, the JLS was moderately correlated in the expected direction with other related workplace characteristics including job control, rewards, community, fairness, and values. Based on correlations of the JLS with many subscales from the Areas of Working Life Survey (AWS), we can conclude that workers who report more health-related leeway also describe other positive aspects of their work environment (autonomy, trust, recognition, etc.). Therefore, offering aging or ill workers more leeway to perform their jobs in their own ways may be part of larger efforts to support and recognize employees. The JLS also showed statistically significant positive correlations with self-efficacy, control, and engagement and negative correlations with fatigue and productivity loss. Correlations of the JLS with work self-efficacy and fatigue remained statistically significant after controlling for job demands and control, suggesting that health-related job leeway is not completely redundant with the more well-studied factor of job control.

Work disability has been described as a complex interaction of factors within the individual, employer, legislative, societal, and healthcare domains [38]. For workers with chronic health conditions, utilizing job leeway to prevent permanent work disability may require trial-and-error attempts to flex specific job tasks, but this requires the organizational support necessary to implement allowable changes. Coordination with coworkers and supervisors may also be necessary to obtain assistance and direction while averting negative impacts to fellow coworkers and to maintain expected levels of service or productivity.

One goal of the authors was to create a scale that would be reliable and valid across a variety of diagnostic groupings. Although our initial sample endorsed many chronic conditions, the predominant categories involved musculoskeletal pain, and sample size limitations prevented a more fine-grained analysis of psychometric properties by diagnostic groupings. Future studies should compare availability and benefits of job leeway for workers reporting musculoskeletal, mental health, and other common chronic conditions.

The construct of job leeway has practical implications with respect to organizational decision-making, for supervisor training, and for facilitating individual-level problem solving and job modification of workers with intermittent or episodic health symptoms. In industries with an aging workforce, providing job leeway may prevent unnecessary work disability or early retirement. For workers, using available job leeway may forgo the need for permanent formal job accommodations or unnecessary periods of disability absence. Educating workers about available forms of job leeway may be one method to provide organizational assistance and to support individual-level problem solving. Training for supervisors might support intermittent job flexibility as a reasonable form of accommodation and clarify policies, autonomy for decision-making, and methods for problem-solving at the working group level [39–41]. Future studies might also evaluate any consistent group-level trends in health-related job leeway by occupation, industry, or other workplace characteristics.

Working from home was the least frequently endorsed item on the JLS, as data from this study were collected prior to the COVID-19 pandemic and many of the workers in this sample were performing essential functions that would not have allowed working from home. For workers with disabilities, telework has been a recognized accommodation strategy [42], but with the large number of those presently working from home or with hybrid working arrangements, telework may also represent a major form of job leeway for many workers for the first time. If home working arrangements continue to be a source of flexibility for many workers, a potential improvement to the JLS would be the addition of more items assessing job leeway in this new context and reality of working from home.

A limitation of the study is the representation of only a few occupations and industries in the test sample; future administrations of the JLS should access broader working populations to re-assess its reliability and validity under more variable working conditions and with more sociodemographic diversity. Also, the JLS assesses only those elements of leeway that are perceived to be available to workers, not those elements that might be most effective for managing symptoms. A third limitation was the cross-sectional nature of the data that limited the ability to test predictive validity of the JLS with longer term occupational outcomes among workers with chronic health diagnoses.

A majority of our initial test sample was female, and this may have implications for measuring job leeway and its organizational benefits. Job flexibility, especially flexible working hours, have been shown to be more highly pursued and valued by female workers [43, 44]. At the same time, female workers perceive less job flexibility compared with their male counterparts [45]. Flexible work arrangements may also have differential impacts on the well-being of male and female workers [46, 47]. The decision-making of employers and supervisors to allow more job flexibility to allow workers to manage intermittent or episodic health problems may vary by gender and have differential outcomes.

Providing more leeway to aging workers may have advantages for employers by reducing turnover, retaining talent, improving worker engagement, and increasing productivity, but these claims require further study. While proactive disability management practices have been associated with improved disability outcomes [48, 49], the business advantages of providing more health-related job leeway to workers needs more study. Future studies should assess the ability of leeway to predict worker retention or prevent early retirement, sickness absence, and permanent disability pensions.

In summary, the 18-item JLS showed initial evidence of reliability and validity in a sample of workers with chronic conditions facing intermittent or episodic functional challenges at work. The JLS measure showed moderate correlations with related workplace constructs and explained unique variance in some outcomes. More research is needed to evaluate the JLS in more representative working populations, but this scale may be helpful to highlight organizational aspects of support and accommodation not commonly appreciated for employer policy making and worker health self-management.

## Data Availability

The datasets generated and analyzed during the current study are available from the corresponding author upon reasonable request.

## Appendix A: The Job Leeway Scale

*Instructions*  We are interested in how much freedom and flexibility you have to modify the way you get your work done when you’re not feeling well.  Please rate your level of agreement with each of the following statements from 0 = completely disagree to 6 = completely agree.

**Table:**

	Completely disagree			Completely agree			
1. When I’m not feeling well, I can rotate between a number of job tasks	0	1	2	3	4	5	6
2. When I’m not feeling well, I can control the pacing of my work	0	1	2	3	4	5	6
3. When I’m not feeling well, I can choose easier job tasks	0	1	2	3	4	5	6
4. When I’m not feeling well, I can choose special tools or equipment that help at work	0	1	2	3	4	5	6
5. When I’m not feeling well, the physical aspects of my job can be altered	0	1	2	3	4	5	6
6. When I’m not feeling well, uncomfortable or stressful aspects of my work can be avoided	0	1	2	3	4	5	6
7. When I’m not feeling well, I can choose between physical and seated tasks	0	1	2	3	4	5	6
8. When I’m not feeling well, I still need to perform my work exactly like everyone else	0	1	2	3	4	5	6
9. When I’m not feeling well, I am able to vary my work	0	1	2	3	4	5	6
10. When I’m not feeling well, I can work from home	0	1	2	3	4	5	6
11. When I’m not feeling well, I can reschedule some of my work activities for another time	0	1	2	3	4	5	6
12. When I’m not feeling well, people can shift responsibilities to help me	0	1	2	3	4	5	6
13. When I’m not feeling well, I can depend on others to help get the work done	0	1	2	3	4	5	6
14. When I’m not feeling well, I can find a more comfortable place to work	0	1	2	3	4	5	6
15. When I’m not feeling well, I can dress more comfortably at work	0	1	2	3	4	5	6
16. When I’m not feeling well, I can take more micro-breaks	0	1	2	3	4	5	6
17. When I’m not feeling well, my job can be flexible	0	1	2	3	4	5	6
18. When I’m not feeling well, my job provides me the leeway I need to get through the day	0	1	2	3	4	5	6
